# Extraction and annotation of human mitochondrial genomes from 1000 Genomes Whole Exome Sequencing data

**DOI:** 10.1186/1471-2164-15-S3-S2

**Published:** 2014-05-06

**Authors:** Maria Angela Diroma, Claudia Calabrese, Domenico Simone, Mariangela Santorsola, Francesco Maria Calabrese, Giuseppe Gasparre, Marcella Attimonelli

**Affiliations:** 1 Department of Biosciences, Biotechnologies and Biopharmaceutics, University of Bari, Bari 70126, Italy; 2 Department of Medical and Surgical Sciences, University of Bologna, Bologna 40138, Italy; 3 Department of Biological Sciences, University of Calgary, Calgary, Alberta, T2N 1N4, Canada; 4 Department of Science, University of Sannio, Benevento 82100, Italy

## Abstract

**Background:**

Whole Exome Sequencing (WES) is one of the most used and cost-effective next generation technologies that allows sequencing of all nuclear exons. Off-target regions may be captured if they present high sequence similarity with baits. Bioinformatics tools have been optimized to retrieve a large amount of WES off-target mitochondrial DNA (mtDNA), by exploiting the aspecificity of probes, partially overlapping to Nuclear mitochondrial Sequences (NumtS). The 1000 Genomes project represents one of the widest resources to extract mtDNA sequences from WES data, considering the large effort the scientific community is undertaking to reconstruct human population history using mtDNA as marker, and the involvement of mtDNA in pathology.

**Results:**

A previously published pipeline aimed at assembling mitochondrial genomes from off-target WES reads and further improved to detect insertions and deletions (indels) and heteroplasmy in a dataset of 1242 samples from the 1000 Genomes project, enabled to obtain a nearly complete mitochondrial genome from 943 samples (76% analyzed exomes). The robustness of our computational strategy was highlighted by the reduction of reads amount recognized as mitochondrial in the original annotation produced by the Consortium, due to NumtS filtering.

An accurate survey was carried out on 1242 individuals. 215 indels, mostly heteroplasmic, and 3407 single base variants were mapped. A homogeneous mismatches distribution was observed along the whole mitochondrial genome, while a lower frequency of indels was found within protein-coding regions, where frameshift mutations may be deleterious. The majority of indels and mismatches found were not previously annotated in mitochondrial databases since conventional sequencing methods were limited to homoplasmy or quasi-homoplasmy detection. Intriguingly, upon filtering out non haplogroup-defining variants, we detected a widespread population occurrence of rare events predicted to be damaging. Eventually, samples were stratified into blood- and lymphoblastoid-derived to detect possibly different trends of mutability in the two datasets, an analysis which did not yield significant discordances.

**Conclusions:**

To the best of our knowledge, this is likely the most extended population-scale mitochondrial genotyping in humans enriched with the estimation of heteroplasmies.

## Background

Mitochondrial DNA (mtDNA) polyploidy is a physiologic trait of human cells and implicates the possibility of the co-existence of different mtDNA genotypes within the same cell, tissue, or individual, a condition known as heteroplasmy. Up to date, quantification of heteroplasmy remains a challenging task in the characterization of mitochondrial variants and a limit for conventional sequencing methods [1]. The advent of Next Generation Sequencing (NGS) technologies has revolutionized the field of genomics, providing the possibility of unprecedented large-scale and high-throughput analyses. Indeed, massive-parallel sequencing implies ultra-deep yields, allowing the quantification of mitochondrial heteroplasmic variants [2,3]. One of the recent applications of NGS is Whole Exome Sequencing (WES), a powerful and quite cost-effective strategy to perform targeted deep sequencing of genomic protein coding regions [4]. Even though the most recent WES protocols include the use of specific baits targeted to mtDNA, the majority of kits currently used is devoted to the enrichment of nuclear-coding DNA, while mtDNA targeting has mostly been neglected [5]. Nonetheless, it was recently demonstrated that the precious information of mitochondrial genotype may be retrieved from off-target DNA in human WES studies, even when designed for nuclear DNA exclusively [6]. It was indeed observed that the overlapping of nuclear probes onto nuclear mitochondrial sequences (NumtS [7]) determines a cross-hybridization of such baits with mtDNA, which in turn is brought along as a 'contaminant' [6]. The natural abundance of the mitochondrial molecules in cells allows to achieve a high read depth, so that a recovery and assembly of the mtDNA genome from nuclear WES studies is indeed feasible [6] together with the quantification of heteroplasmy wherever the mitochondrial genome is sufficiently covered.

The relevance of estimating mitochondrial heteroplasmy is further highlighted by the fact that mtDNA mutations exert their phenotypic effect above a certain mutation load threshold [8,9], which may vary depending on the type of change [8,10] and tissue. Indeed, several studies demonstrated that mtDNA mutations are functionally recessive until the mutant load exceeds a specific threshold and leads to a biochemical dysfunction [8,9,11]. In fact mitochondrial mutations are largely involved in various diseases, aging and cancer [12]. In addition, the finest quantification of heteroplasmy among familiar lineages is helpful for forensic studies [13] and to better understand mechanisms of intergenerational segregation, especially in the case of maternal transmission of mutations predisposing to mtDNA disorders [14].

Even though since 1995 it is known that heteroplasmy in normal individuals may not be a rare biological status [15], only recent surveys on mitochondrial genotyping and heteroplasmy annotation with deep sequencing have revealed that in normal human cells a widespread heterogeneity of mtDNA variants co-existence occurs in healthy subjects and varies among tissues [1]. Moreover, a condition of 'universal heteroplasmy' was depicted by Chinnery and colleagues in their recent work [16] in which they observed, by using high-throughput technologies, the presence of very low-level heteroplasmic variants in related and unrelated individuals, likely due to inherited or somatic events, not predicted to be pathogenic.

So far, consistently with the limited sensitivity of strategies and with the restricted population sampling available for mitochondrial genotyping, commonly used mitochondrial portals and databases [17,18] do not report heteroplasmy values for the mutations/variants whose fraction has been reported in literature. However, while such information is lacking in databases, nucleotide and amino acid sites variability is at least available within HmtDB [18] as a valuable information that contributes both to the definition of haplogroups and to the recognition of private variants or mutations with a potential pathogenic role.

In this work we used a pipeline previously provided [6], that we further implemented for insertions and deletions (indels) calling and applied to the *in silico *extraction and characterization of mitochondrial DNA from nuclear WES studies, comprising 1242 exome samples of the 1000 Genomes project [19] belonging to 19 different human populations. 1000 Genomes Project WES studies represent one of the widest nuclear genomic resources available to the scientific community, yet strikingly, up to now, mtDNA genotyping has been disregarded, despite the important contribution the small mitochondrial genome provides to the cell homeostasis. We were able to recover mtDNA from all WES studies and to assemble the complete mitochondrial genome for the majority of analyzed samples. High quality metrics were used to filter mismatches and indels and related heteroplasmy values of filtered variants were recorded. We enriched these data with a functional characterization, firstly detecting haplogroup and non-haplogroup defining sites. Variability estimation reported in the human mitochondrial database and predictions of pathogenicity [20-22] were also applied to non-haplogroup defining variants, revealing an intriguingly heterogeneous scenario among populations. We also performed a comparison between lymphoblastoid cell lines (LCLs) and blood-derived mitochondrial DNA isolated from the group of available samples, in order to assess the possible presence of genotype differences among the two different DNA sources, as previously tested for nuclear DNA [23-25]. Finally, in line with the recent finding of the widespread distribution of heteroplasmy among healthy individuals, we found a consistent enrichment, among all 19 human populations screened, of very low-levels heteroplasmies, mostly unshared between subjects and associated with very low variability values reported in HmtDB, which more likely represent a bulk of private variants carried by each individual.

## Methods

### Exome samples

We surveyed Whole Exome Sequencing (WES) datasets related to 1242 individuals, available through the 1000 Genomes project site [26]. These individuals included 827 samples of the 1000 Genomes Phase 1 data collection, while the remaining 415 were additional individuals (Additional File 1), listed within the 2012-05-22 exome alignment index [27], which comprised all the exome samples aligned by the Consortium until then. Our dataset included 625 females and 617 males, from 19 different populations in total (Table 1). The set of samples was selected considering different criteria: sequences were all Illumina paired-end reads whose mates were both mapped onto the references (Genome Reference Consortium Human Reference 37, GRCh37/hg19 [GenBank:GCA_000001405.1] for nuclear reads, and the revised Cambridge Reference Sequence, rCRS [GenBank:J01415.2] for mitochondrial reads), using the Burrows-Wheeler Aligner (BWA) [28]. Two different platforms for whole exome capture were used for the samples analyzed: SeqCap EZ Human Exome Library v1.0 and v2.0 from NimbleGen (for 515 samples) and SureSelect All Exon V2 Target Enrichment from Agilent (for 723 samples) [19], whereas for four samples it was not possible to retrieve any information. All the information about our 1242 samples was available through the 1000 Genomes web site [26].

**Table 1:** Features of the 19 populations analyzed in this study.

Population Code	Population	N. of Samples	Mitochondrial Mean Coverage	Mitochondrial Mean Per Base Depth
**ACB**	African Caribbean in Barbados	58	47.34	48.15

**ASW**	African Ancestry in Southwest USA	51	99.89	373.39

**CDX**	Chinese Dai in Xishuangbanna, China	78	97.64	47.44

**CEU**	Utah residents with ancestry from northern and western Europe	67	96.87	311.71

**CHB**	Han Chinese in Beijing, China	73	99.05	142.86

**CHS**	Han Chinese South, China	90	88.16	40.87

**CLM**	Colombian in Medellin, Colombia	48	93.14	234.13

**FIN**	Finnish in Finland	81	85.35	96.45

**GBR**	British From England and Scotland, UK	69	96.12	269.39

**GIH**	Gujarati Indians in Houston, Texas, USA	77	47.3	41.54

**IBS**	Iberian populations in Spain	58	83.67	106.6

**JPT**	Japanese in Tokyo, Japan	74	94.02	217.41

**KHV**	Kinh in Ho Chi Minh City, Vietnam	75	68.05	25.12

**LWK**	Luhya in Webuye, Kenya	25	93.71	256.66

**MXL**	Mexican Ancestry in Los Angeles, California, USA	58	99.91	409.61

**PEL**	Peruvian in Lima, Peru	47	63.61	33.67

**PUR**	Puerto Rican in Puerto Rico	70	82.13	87.29

**TSI**	Toscani in Italia	62	98.36	328.96

**YRI**	Yoruba in Ibadan, Nigeria	81	94.92	214.86

For each population the number of samples we considered and the mean coverage and depth of mitochondrial genomes we extracted from exome data, are reported. Coverage and depth were estimated as average of scores in each sample belonging to the same population. Mean coverage of assembled mitochondrial genomes ranges from 99.91 (Mexican Ancestry in Los Angeles, California, USA) to 47.30 (Gujarati Indians in Houston, Texas, USA). Mexicans show also the highest mean per base depth (409.61), while the minimum depth score is for Kinh (Ho Chi Minh City, Vietnam) mitochondrial genomes (25.12).

### Mitochondrial sequences recovery from 1000 Genomes WES studies, variant calling and heteroplasmy assessment

We adopted a pipeline previously published [6] in order to recover mtDNA from off-target sequences of WES studies, removing reads similar to NumtS (Nuclear Mitochondrial Sequences) [7] and maintaining only reads with a univocal mapping onto the mitochondrial genome. Original mitochondrial BAM (Binary SAM, Sequence Alignment/Map format) files from the 1000 Genomes Project were first converted in fastq files using the *SamToFastq *module of the Picard suite of tools (v.1.68) [29] to apply the abovementioned pipeline, which requires the usage of SAMtools (v.0.1.19) [30], GSNAP (version 2012-01-11) [31] and python 2.7.1 [32]. After read mapping on the mitochondrial genome, using rCRS as reference sequence, the *MarkDuplicates *module of the Picard tools was applied on obtained SAM files in order to remove PCR duplicates. Duplicates filtering was just recommended in the adopted pipeline, but we considered this step crucial for a correct allelic quantification in our analysis. We extended variant call analysis also to indels, since they were not completely taken into account by the pipeline we used. In particular, we observed that it was targeted only for the detection of homoplasmic deletions, disregarding heteroplasmic indels. Moreover, the tool previously developed did not automatically quantify heteroplasmic fractions (HF) that we instead implemented in our system. We thus implemented a workflow in python programming language to parse the SAM file CIGAR string [30] associated to each pairwise aligned read, holding information on indel events. Mismatches were instead analyzed directly from the *mtDNAassembly-table.txt *output of the adopted pipeline. Filtering of insertion events was performed using quality score (QS≥25) and read depth (rd≥5) cut-off values *per *position previously adopted [6], whereas the goodness of a deletion event was evaluated on the basis of its 5bp-long upstream and downstream flanking region. Indeed, a median QS≥25 and rd≥5 were required for the flanking regions to consider such deletion in the genome assembly. HF was assessed as the fraction of the variant read depth onto the total mitochondrial read depth of the same position (for mismatches and deletions) or of the 5' flanking position (for insertions).

The adopted pipeline allowed also to produce a consensus mitochondrial sequence for each individual, including only mismatches equal or above a HF threshold of 0.75, otherwise the corresponding IUPAC character for single nucleotide variants was reported [6]. This consensus was used for the haplogroup assignment and for the subsequent analysis of pathogenicity linked to non-synonymous mismatches considering also all those variants under the fixed threshold annotated with IUPAC code.

All mitochondrial unique variants detected in this study are reported in a BED (Browser Extensible Data) table (Additional file 2), generated from a VCF file which is the output of the python script we developed for the detection of indels and heteroplasmic fraction of mitochondrial variants. The python script and the VCF file (reporting all the detected mitochondrial variants integrated with the related heteroplasmic fractions) are available upon request.

### Haplogroup assignment

Haplogroup assignment of 943 mitochondrial consensus sequences was performed using the stand-alone *mt-classifier *tool, implemented in HmtDB [33], which uses the Reconstructed Sapiens Reference Sequence (RSRS) [34] as mitochondrial reference.

The samples were chosen among the best assembled mitochondrial genomes within our dataset, harboring less than 500 gaps in their sequence. The *mt-classifier *version used refers to the Phylotree [35] mtDNA tree build 15.

### Mitochondrial reference databases

Variants identified within the analyzed dataset were compared with those already annotated within HmtDB [18] and Mitomap [17].

Site variability values calculated on mitochondrial nucleotide multi-aligned sequences from nearly 10,000 healthy individuals, were downloaded from HmtDB web site [33]. Variability scores contributed to the recognition of private variants or mutations with a potential pathogenic role: they ranged from 0 to 1, where low variability values may be suggestive of a novel haplogroup-defining variant or a rare disease-linked mutation, while high variability scores were owned by common alleles within genomes stored in HmtDB.

Mitochondrial coding and control regions point mutations with reports of disease-associations were available in Mitomap [36].

### Pathogenicity predictions

Pathogenicity predictions on variants identified within the analyzed dataset were estimated using MutPred [20], Polyphen-2 [21] and SNPs&GO [22] online software, producing 6 different scores and related prediction classes for each variant. Pathogenicity values, ranging from 0 to 1, allowed discriminating between potentially pathogenic and neutral mutations. Each of the abovementioned software presents a specific threshold of pathogenicity. We considered 0.70 as threshold for high pathogenicity score by MutPred as suggested by Pereira *et al*. [37] and "disease" prediction class by the other ones.

### Hierarchical clustering analysis

A hierarchical clustering based on Euclidean distance estimation was performed to evidence shared classes of heteroplasmy within individuals with the aim to identify a possible clusterization of samples belonging to the same population sharing a similar number of variants referred to the same heteroplasmic range. The heatmap representation was used to visualize hierarchical clustering by rows and columns, using a matrix of numbers of variants belonging to 11 different classes of heteroplasmy, as raw input data. The *maPalette *function from the *marray *R package version 3.0.1 was used to define heatmap colors, while the *heatmap *R function was used to draw the heatmap.

### Lymphoblastoid cell lines and blood subsets

We identified 60 potential blood samples among our 943 best assembled mitochondrial genomes and chose 60 potential LCL samples on the basis of Epstein-Barr virus (EBV) coverage values provided by the Consortium [38]. Indeed an EBV coverage equal to 0 might indicate blood derived DNA, while LCL samples were selected among those with the highest EBV coverage (in this case, higher than 400).

### Statistical analyses

Spectra of variants (insertions, deletions, mismatches) within blood and LCL samples were compared with one-tailed and two-tailed Student t-tests. Fisher's test was applied to the distributions of blood and LCL heteroplasmic variants within mitochondrial loci and also to the distributions of variants within the same datasets *per *class of heteroplasmic fractions.

## Results

### Coverage and quality of assembled mitochondrial genomes

We downloaded aligned exome data (as BAM files) related to 1242 individuals of the 1000 Genomes Project from the public repository [39]. Sequence reads were extracted from the BAM files and re-aligned to the human reference genomes to assemble mitochondrial genomes for all the samples by applying Picardi's pipeline [6].

The first step of our analysis was the assessment of quality and coverage of the reconstructed mitochondrial genomes. We found that 76% of them showed a nearly complete assembly (mitochondrial genome coverage >97%). The robustness of the computational strategy we used was highlighted by the reduction of the read count (an average of 30% *per *sample) mapped to the reference mitochondrial genome with respect to the original annotation by the 1000 Genomes Consortium. Two different factors contributed to the reduction of the original number of reads, namely PCR duplicates removal and, particularly, NumtS filtering [7], disregarded in the mapping step performed by the Consortium.

The mitochondrial assembly showed different mean coverage values within the 19 analyzed populations (Table 1): they ranged from 99.91% to 47.30% with respect to the entire mitochondrial genome. Mean *per *base depth was also widely variable, ranging from 409.61 to 25.12. Detailed data about mitochondrial genome coverage of each sample are reported in Additional File 1.

Coverage and quality score (QS) statistics were performed for each mitochondrial locus by estimating median read depth and QS (Additional File 3 and Additional File 4). As already reported [6], a better efficiency in mitochondrial reads extraction was obtained with the Agilent enrichment kit, as suggested by comparing the highest mitochondrial depth values obtained within the same locus (*MT-ND6*) through the Agilent (274.57X) and the NimbleGen samples (55.67X). Furthermore, Agilent mitochondrial reads showed slightly higher mean QS than NimbleGen (34.62 vs 32.22), with the maximum and the minimum QS observed within the same loci, *MT-TS2 *(35.82 vs 33.13) and *MT-TR *(34.30 vs 31.47) respectively. Intergenic regions within Agilent and Nimblegen samples presented the same trend of QS and coverage values as of D-Loop and coding regions (data not shown).

To verify the robustness of our protocol, we further selected a subset of 28 samples with a mitochondrial genome coverage >99%, which were previously analyzed with both low coverage Whole Genome Sequencing (WGS) [40] and Exome Sequencing projects by the Consortium [19]. The dataset included 2 samples for 14/19 populations. None of the analyzed exome samples was found within the low coverage dataset for the remaining 5 populations. We considered the subset of 28 samples (indicated as "Low Coverage Control" in Additional File 1) with the aim of identifying possible disagreements about variants detected by the Consortium and our pipeline. We were able to detect up to 93.53% of mitochondrial variants per sample reported within the low coverage Variant Call Format (VCF) file generated by the Consortium (Additional File 5), while 45/89 were missed with exome samples as they were not sufficiently covered. Moreover, on average 3 variants *per *sample were not identified within exome samples, although their positions were sufficiently covered, whereas we were able to find more than 10 variants per sample not previously annotated with low coverage analysis by the Consortium. The latter were all heteroplasmic and about the 70% presented a HF<0.10, proving the sensitivity of our pipeline.

### Haplogroup assignment of reconstructed mitochondrial genomes

The haplogroup assignment procedure helped to test the quality of reconstructed mitochondrial sequences, since the geographical areas of sampled individuals were known. To this aim, we selected 943 out of 1242 samples (Methods section), which were analyzed by applying the *mt-classifier *implemented in HmtDB [18]. Generally the predicted haplogroups seemed to converge with individual ethnicity.

About 93% of the 943 reconstructed sequences presented a prediction reliability P_Hg >90%, defined as the highest fraction of Nph (recognized SNPs in the sequence which define haplogroup (Hg) according to Phylotree (ph) classification [35]) over the total number of the haplogroup-defining expected sites (N_ph_exp_) [18]. This high percentage of true-positive haplogroup-defining variant sites confirmed the high sensitivity of our method. Assigned haplogroups are reported in Additional file 1.

### Indels and mismatches recognition, mapping and *in silico *validation

**Figure 1:**
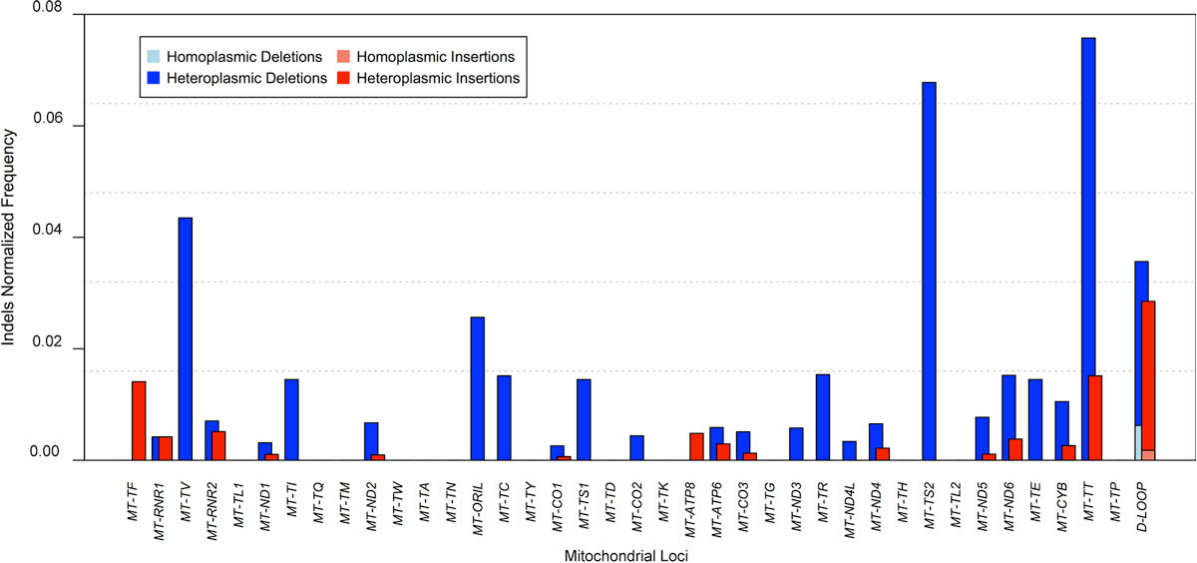
**1000 Genomes mitochondrial indels distribution**. The analysis of 1242 Illumina samples allowed to identify 149 deleted and 66 inserted mitochondrial positions, mostly heteroplasmic. The ratio between the number of homoplasmic and heteroplasmic indels and the length of mitochondrial loci is reported (normalized indels). The distribution of insertions and deletions within the mitochondrial genome shows peaks of indels ratio within the *D-loop*, while a lower number is present within coding regions.

An accurate analysis of indels and mismatches was carried out on our mitochondrial dataset, considering the role played by these mutation events in healthy and pathological samples [41,42]. In considering the values reported below it is important to take into account that the same event may be observed in diverse samples in a different heteroplasmic status. Two hundred and fifteen unique mtDNA indel events (149 deletions and 66 insertions), mostly heteroplasmic, were firstly recognized within the 1242 mitochondrial genomes analyzed (Additional File 6), mapped onto the mitochondrial genome (Figure 1) and then compared with indels already annotated in mitochondrial databases [17,18] (Additional File 6).

About 56% samples harbored at least one deletion and 64% presented an insertion (Additional File 7). The most common events were single-base insertions (in 69% cases) and deletions involving up to 3 bases (in 50% cases; data not shown). However their distribution normalized to the length of each mitochondrial locus showed high peaks of indels ratio within the *D-loop*, while a lower number was present within coding regions, where frameshift mutations may be deleterious (Figure 1).

Interestingly, a certain amount of deletions was detected also within genes for transfer RNA. Intergenic regions, not shown in Figure 1, included 6 heteroplasmic insertions, 3 heteroplasmic deletions and 2 homoplasmic deletions.

The majority of the indels identified (72.09%) occurred within homopolymeric stretches (Additional file 8), defined as regions with the same nucleotide in at least two adjacent positions. The shortest homopolymers harbored the highest number of indels, although 5-bases stretches presented high levels of both deletions and insertions too, with the exception of G-stretches. This result was quite expected considering the homopolymeric nucleotide compositional bias of the mitochondrial genome, and that almost all of the homopolymers (70%) within the mitochondrial reference genomes (rCRS and RSRS) are represented by two-bases stretches. Moreover, up to 25% of the identified indels mapped within mitochondrial tandem repeat regions, where the onset of mutational events is favored [43,44].

The set of indels found was compared with those already annotated in mitochondrial databases [17,18], validated through traditional sequencing methods, discriminating those found in healthy individuals from those in diseased patients. About 20% indels observed in our samples matched with those reported in HmtDB within healthy samples dataset and nearly 8% were annotated in patient genomes (Table 2). As expected based on the NGS technologies power in detecting also low-level heteroplasmy [1,45,46], the remaining 80% highlights that there was a clear-cut prevalence of novel deletions and insertions, which mostly spanned non-coding regions, even though coding regions were not completely devoid of them. Interestingly, a single base insertion at the position 3229 (the 3'end nucleotide of *16s rRNA*), single base deletions in 5747 (*OriL*, origin of light-strand replication) and 11032 (*MT-ND4*), and the extended deletion in 303-305 (*D-Loop*) were present only in patients, but not yet associated to any disease [18]. With respect to the 18 insertions and 15 deletions reported in Mitomap, some of them are associated to cancer or other diseases, *i.e*. a set of *D-loop *mutations (*m.514insCG *found in head/neck tumor, *m.568insC(n) *found in multiple tumor types, *m.16189insT *found in bladder tumor, *m.310delT *found in breast tumor), three indels events in the mitochondrial gene encoding 12S rRNA, *MT-RNR1*, (*m.956insC, m.956delC, m.960delC*, which are possibly DEAF-associated) and one deletion in the gene encoding for tRNA threonine, *MT-TT*, (*m.15940delT *found in multiple myeloma) [17].

**Table 2:** Summary related to indels and mismatches detected within the 1242 analyzed mitochondrial genomes

Events	% Homoplasmic	% Annotated in Healthy	% Annotated in Patients	% Heteroplasmic	% Annotated in Healthy	% Annotated in Patients
Indels	6.05	15.38	0.00	98.60	21.23	7.91
Mismatches	64.69	88.48	76.36	68.39	62.75	53.65

Statistics about homoplasmic and heteroplasmic mitochondrial events are here reported. Events are discriminated between indels and mismatches. The percentages of homoplasmic and heteroplasmic observed variants annotated in healthy and patients genomes stored in HmtDB [18] are reported. A fraction of variant sites was found as homoplasmic in any samples and heteroplasmic in others.

**Figure 2:**
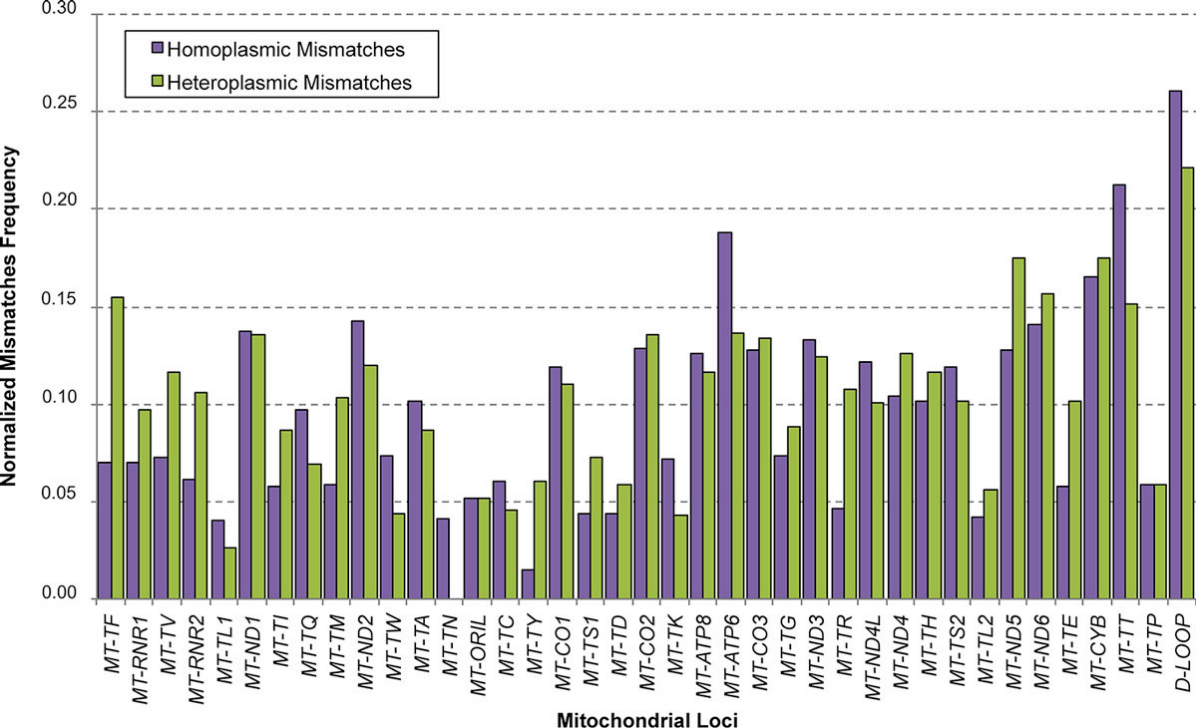
**1000 Genomes mitochondrial mismatches distribution**. The ratio between the number of mismatches and the length of mitochondrial loci (normalized mismatches) is reported. Variant distributions are rather homogeneous among mitochondrial loci, without significant dissimilarities between the two types, with high peaks into non-coding regions.

Also 3407 single base mismatches were detected within our samples, involving 3289 unique positions (Additional File 9), with an average of about 25 mismatches per sample. They were discriminated between 2330 heteroplasmic and 2204 homoplasmic alleles, whose normalized distributions are shown in Figure 2. Such distributions are rather homogeneous among mitochondrial loci, without significant dissimilarities between the two types, with high peaks into non-coding regions. As well as for indels, we found about 40% mismatches positions spanning homopolymeric stretches and only 1% within mitochondrial variant nucleotide tandem repeats (data not shown). The majority of single-base homoplasmic variants (88.48%) was already described in healthy individuals [18] and 62.75% heteroplasmic ones were annotated in public databases in a homoplasmic state (Additional File 9). A large fraction of hetero/homoplasmic mismatches (more than 50% and 76%, respectively) was also found in patients. Statistics related to indels and mismatches are summarized in Table 2.

### Heteroplasmy analysis

**Figure 3:**
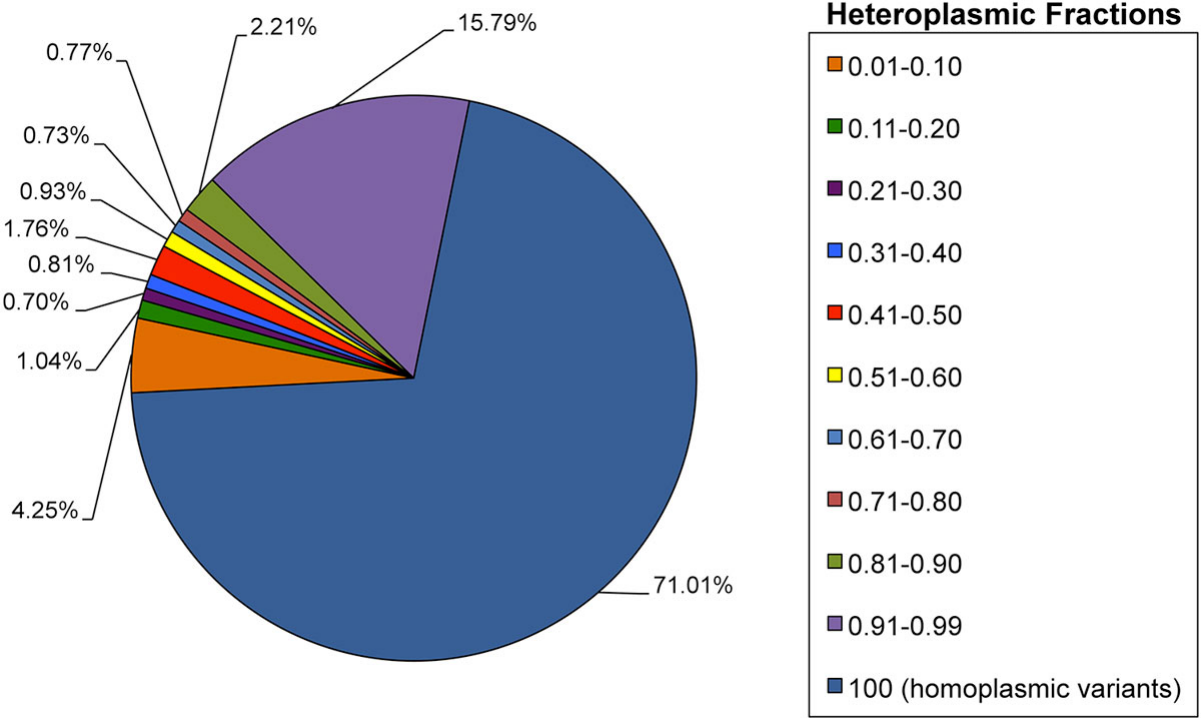
**Enrichment of heteroplasmic fractions within the dataset**. Variants frequency was estimated for eleven ranges of heteroplasmic fractions (HF). The ratio between the mean number of variants for each cluster and the mean number of variants in each sample highlights that homoplasmic variants represent the largest slice with respect to the whole set of alleles in an individual. With respect to the degree of heteroplasmy, there is a substantial preponderance of homoplasmic variants (HF = 1.00), quasi-homoplasmies (0.90-0.99) and low-level heteroplasmies (0.01-0.10).

We detected on average 20 homoplasmic and 8 heteroplasmic variants *per *sample (Additional File 7). Frequency of variants was estimated for eleven ranges of heteroplasmic fractions (HF) (Figure 3). Considering the mean number of variants for each range of heteroplasmy and the mean number of alternative alleles in each sample, homoplasmic variants represented the largest slice with respect to the whole set of alleles in an individual. There was a substantial preponderance of homoplasmies (HF = 1.00), quasi-homoplasmies (defined as 0.90<HF<0.99) and low-level heteroplasmies (0.01<HF<0.10), suggested also by a hierarchical clustering analysis (Additional File 10). We did not observe correlation between clusters of heteroplasmic variants and sample geographical origin.

### Pathogenicity analysis of private mutations

After haplogroup assignment, we isolated more than 2500 variants not defining the individual haplogroup, then functionally annotated. Predictions of pathogenicity were carried out on the subset of 543 non-synonymous variant positions using MutPred [20], Polyphen-2 [21] and SNPs&GO [22] tools with the aim of identifying potentially damaging alleles and their spread within the 19 populations (Additional File 11 and Additional File 12). All the 543 missense mismatches were classified according to the ranges of heteroplasmy, so that 339 were homoplasmic and 309 heteroplasmic. Above all, *MT-ND5 *appeared to harbor the highest frequency of non-synonymous changes. Occurrence of variants in the reconstructed mitochondrial genomes was not exclusive for a particular world area, but generally a variable number of samples belonging to different populations shared the same homoplasmic and quasi-homoplasmic/highly heteroplasmic variants, while low-level heteroplasmies were detected only in single individuals and may hence be likely considered personal mutations. For example, the *m.13105A>G *event was shared by 106 samples belonging to 14 populations, with the lowest HF equal to 0.87 (Additional File 11). We focused only on 289 variant sites (148 found in a homoplasmic state, 183 as heteroplasmic) predicted as probably pathogenic by at least one of the above mentioned software (Additional File 12) and interestingly they all showed very low variability (mean value = 0.02 for homoplasmic mutations, 0.03 for the heteroplasmic ones). Among potentially damaging events, the lowest HF value was 0.25 (Additional File 11). The most shared variants within this subset were all in a homoplasmic/highly heteroplasmic state. Only 14 non-shared mismatches were predicted as damaging mutations by the three software, and among them the *m.3946G>A *was also annotated in Mitomap as associated to MELAS (Mitochondrial Encephalomyopathy, Lactic Acidosis and Stroke-like episodes). They all presented a low variability value (<0.20) and a HF ranging from 0.29 to 1.00.

The complete list of mitochondrial unique variants identified is reported in Additional File 2.

### Analysis of LCLs and blood samples

**Figure 4:**
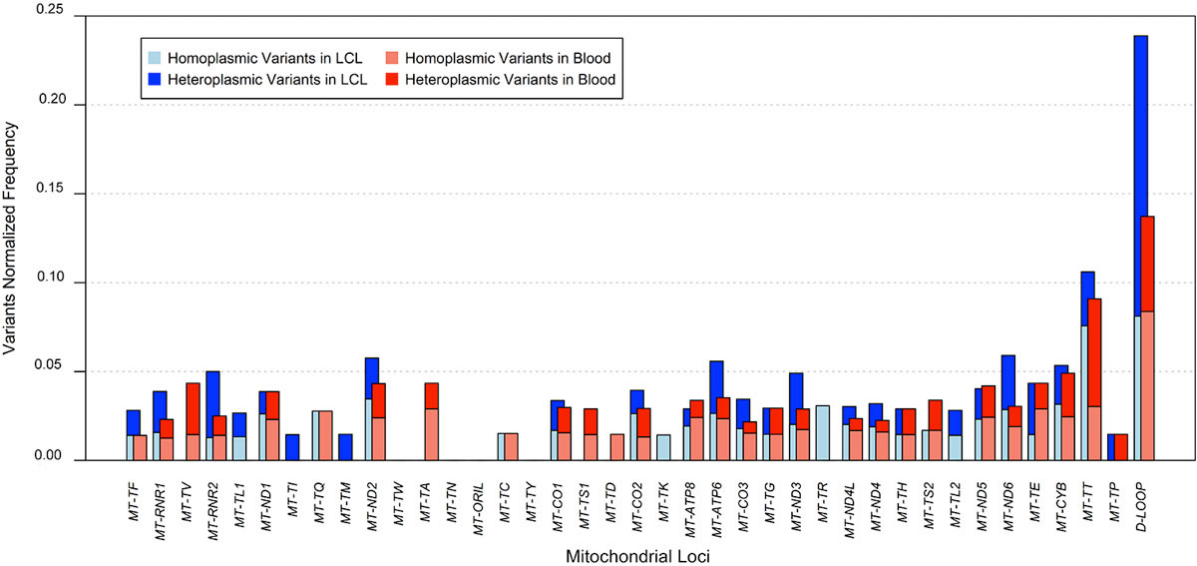
**Distribution of homoplasmic and heteroplasmic variants in LCL and blood subsets**. Frequencies of homoplasmic and heteroplasmic variants were normalized to the length of mitochondrial loci, in order to eliminate length biases. Variants distribution across mitochondrial loci shows that generally heteroplasmic and homoplasmic mutations occur in the same loci, and this is verified in both datasets. A statistical relevant difference between the distributions of variants within the two datasets was found for heteroplasmic (p < 0.01), but not for homoplasmic variants.

As previously observed for nuclear variants [23-25], we hypothesized that lymphoblastoid cell lines (LCLs) might have a higher rate of mitochondrial mutations than blood. Similarly to what already performed for nuclear variants [23-25], we attempted to identify relevant qualitative and quantitative gaps between variants found in 120 out of our 1242 samples, among which 60 were supposed to be derived from blood and 60 from LCLs (Methods). Samples were not related in families, except for one CEU trio (NA12878, NA12891, NA12892), and were representative of 18/19 populations. We first considered variants occurrence within the two datasets and their localization in the mitochondrial genome, discriminating heteroplasmic and homoplasmic sites (Figure 4). Frequencies were hence normalized to the length of mitochondrial loci, in order to eliminate biases related to length. The distribution of variants along mitochondrial loci showed that generally heteroplasmic and homoplasmic mutations occurred at the same loci, which was verified in both datasets. A statistical relevant difference between mitochondrial loci distributions of variants within the two types of samples was found for heteroplasmic (p < 0.01), but not for homoplasmic variants (Figure 4). The total number of variants was estimated within the two subsets, discriminating also between indels and mismatches and considering their heteroplasmy status (see Additional File 7). Minimal statistically relevant differences were detected between LCLs and blood derived DNA especially in relation to indels (p < 0.05).

**Figure 5:**
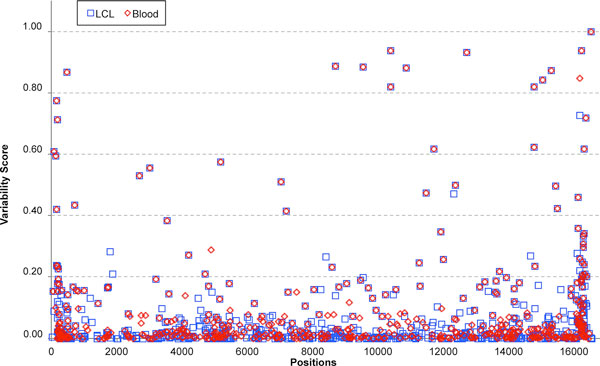
**Variability profile of blood and LCL genomes**. Variability values estimated on nearly 10,000 mitochondrial nucleotide multi-aligned sequences, available in HmtDB web site [33] are reported versus the variant positions observed in both blood and LCL samples. The trend is similar for both datasets: almost all of the variant positions display a very low variability (<0.20). Positions showing high variability, *i.e*. those which were common in the genomes within HmtDB, were few and represented the 25% of total shared variants between the two datasets, while the majority of non-shared variants (about 98%) displayed low variability.

Variant sites observed in the two different DNA sources were associated with variability values estimated on nearly 10,000 complete mitochondrial genome sequences [33] (Figure 5). The trend was similar for blood and LCL samples: almost all of the variant positions displayed a very low variability (<0.20) (Table 3). Positions showing high variability, *i.e*. those which were common in the sequenced genomes available at HmtDB [18], were few and represented the 25% of total shared variants between the 2 datasets, while the majority of non-shared variants (about 98%) displayed low variability. Among low variability sites (<0.20), we found only a minimal numerical difference in low-level heteroplasmy variants (HF<0.10) enriched in LCLs, albeit not supported by statistical significance (Table 3).

**Table 3:** Number of variants within LCL and blood subsets, sorted by heteroplasmic fraction and variability score.

**Variability**	**Sample**	**HF<0.10**	**0.11<HF<0.75**	**0.76<HF<0.99**	**HF = 1**	**F-test**

≤0.20	LCL	66	120	214	783	0.90

≤0.20	blood	22	119	260	820	0.90

>0.20	LCL	3	6	74	489	0.39

>0.20	blood	2	8	49	542	0.39

The highest number of positions shows low variability (≤0.20), and although no statistically significant difference was observed between the LCLs/blood overall distributions of variants, an enrichment in low-level heteroplasmies was detected in LCLs low-variability variants.

In fact, upon analyzing LCLs/blood heteroplasmy levels (Table 4), we confirmed a LCLs trend to accumulate low-level heteroplasmic variants (69 vs 24 in blood samples), although a comparable average number of heteroplasmic variants per sample was reported for both LCL and blood samples (7.42 for LCLs, 7.33 for blood samples). About 25% variants were shared by both subsets and in 80% cases they were homoplasmic.

**Table 4:** Number of heteroplasmic and homoplasmic variants within blood and LCL samples, sorted by heteroplasmic fraction

Heteroplasmic Fraction	N. of Variant Positions in Blood Samples	N. of Variant Positions in LCL Samples
< 0.10	24	69
0.11-0.20	14	28
0.21-0.30	9	10
0.31-0.40	10	19
0.41-0.50	28	27
0.51-0.60	22	19
0.61-0.70	18	13
0.71-0.80	43	18
0.81-0.90	80	42
0.91-0.99	212	238
1.00	1362	1272

A clear enrichment in very low-level heteroplasmic variant positions within the whole LCL dataset (69 vs 24 in blood samples) may be observed.

## Discussion

In this paper we report the results obtained by capturing human mtDNA sequences starting from 1000 Genomes Whole Exome Sequencing (WES) data specifically targeted to nuclear genes. WES has been commonly used with clinical aims since its first application [47], although this technology has been also implemented in evolutionary comparison of genomes [48]. Even though the most recent WES protocols include the use of specific baits targeted to the whole mitochondrial genome as well as the MitoCarta set of nuclear genes [49], the probe sets used in the majority of previously published WES studies excluded such baits, hence mtDNA sequences have been considered by-products and thereby neglected.

For the first time, we here recovered the overlooked mitochondrial information and reconstructed a large number of human mitochondrial sequences from off-target exome data, thanks to the availability of population-scale sequence datasets offered by the 1000 Genomes Project [19]. This was possible through the application of a bioinformatics pipeline aimed at assembling mitochondrial genomes from exome data and then at annotating insertions, deletions and mismatches, accompanied by their heteroplasmy fraction, a series of tasks that were not feasible so far and that are necessary when dealing with mtDNA data. Overall we managed to extract 1242 assembled mtDNA, testing the validity of the protocol on the basis of coverage and quality data of the mitochondrial genomes that were generated. Further evidences of the robustness of our method were provided by the accurate haplogroup assignment, which reflected individual ethnicity, and by the comparison with the set of variants within 28 samples selected from the mtDNA VCF of low-coverage whole genome sequencing data [40] generated by the Consortium. It has to be underlined that a fraction of mitochondrial variants (6.47%) annotated in the low coverage report was not identified by our protocol. This discrepancy may be likely due to variant artefacts as a consequence of WES probes overlapping NumtS, which were not filtered out in the 1000 Genomes project. On the other hand Whole Genome Sequencing protocols ensure the availability of probes mapping on the entire mitochondrial genome, therefore some missing variants derived from the exomes analyzed are linked to an incomplete covering of any mitochondrial genomes. Another possible source of discrepancy may be represented by sequencing errors ruled out by our protocol as associated to quality scores lower than the minimum threshold required for variant calling. The analysis of the reconstructed mitochondrial genomes highlighted a widespread distribution of polymorphisms in healthy samples. Noteworthy, a parallelism may be observed between the enrichment in damaging and probably damaging rare variants within nuclear low frequency alleles of the 1000 Genomes Low Coverage [50] and Exon Pilot Projects [51], and the numerous group of mitochondrial pathogenic predicted alleles [20-22] and mutations with a confirmed disease association [17,18], detected in our dataset.

In fact, Exon Pilot rare variants [51] observed among continents highlighted a reduction in the degree of allele sharing, as well as a very low variability was associated to homoplasmic/heteroplasmic mitochondrial variants we predicted as damaging, that were mainly observed in sparse unrelated individuals. Although low variability may be affected by a bias in the world population sampling, this finding may suggest that, similarly to the nucleus, negative selection is acting on these mitochondrial sites.

Furthermore, similarly to the results on nuclear DNA reported by Xue *et al*. [50], a possible explanation of the presence of pathogenic mitochondrial variants in healthy samples in a heteroplasmic condition below the specific threshold for the onset of the disease [8], at least at the time of recruitment, may be linked to the age of disease onset or to an incomplete penetrance due to the absence in the subject of other factors contributing to the disease; a further explanation may be linked to the presence of erroneous annotations of disease-associated variants in databases. Indeed only a few variants are currently annotated as unequivocally disease-causative, *i.e*. mainly Leber's Hereditary Optic Neuropathy (LHON) and Mitochondrial Encephalomyopathy, Lactic Acidosis and Stroke-like episodes (MELAS) mutations [17], while there is a high degree of uncertainty about the correct disease association for most of the mitochondrial mutations reported in literature especially since most of the time functional studies are lacking. Moreover, no information about the level of heteroplasmy is reported in mitochondrial databases and this is becoming an urgent need, considering the relationship between heteroplasmy threshold and clinical manifestation of the disease [8,11] and the ever increasing power of NGS technology in detecting also minor alleles in heterogeneous pools of variants [1,16,45,46]. As recently shown and in line with our findings, a 'universal heteroplasmy' of low-level heteroplasmic variants is present in healthy humans [16], which may not be disregarded. In this population-scale scenario of variety, a common event recorded within our dataset is length heteroplasmy, that is the coexistence of different lengths of the same homopolymeric stretch within the same individual, mainly occurring within mtDNA control regions and present at a lower frequency in the coding regions. Although coding regions are highly populated with homopolymeric stretches, the lower occurence of length heteroplasmic variants may be due to a negative selection process acting to avoid function disruption.

As general concerns were expressed about the use of LCLs-derived DNA for sequencing studies [52], we further sought differences in LCL versus blood sample subsets. To the best of our knowledge, no previous study showed reports on LCLs and blood mtDNA comparison. Although in our analyses we lacked paired samples of blood and LCLs from the same subject, we disposed of numerous groups of blood and LCL samples from several unrelated individuals (except for one trio), however sufficient to identify qualitative and quantitative gaps between the two, considering that EBV transformation may result in low-level generation of *de novo *mutations [23-25]. Several works on whole exome/genome sequencing of LCLs/blood were all in agreement that the great majority of variants found in paired samples is shared (99% at least) [23-25], except for a minimal percentage of *de novo *variants, also found in low-passages LCLs [24]. A comparable result was observed in our mitochondrial blood/LCL samples: the average number of heteroplasmic variants *per *type of sample was the same (about 7), even though we solely observed a difference in the numerical distribution of heteroplasmic variants along mitochondrial loci (Figure 4). However 80% variants shared by both datasets (25% of all the detected variants) were homoplasmic and the only numerical, albeit not statistically significant discrepancy was recorded for very low-level heteroplasmic alleles (<10%), mostly enriched in LCLs. Moreover, low variability sites abound among non-shared variants, which belong to the low-level heteroplasmy range of variants observed also in blood samples, likely a more reliable DNA source than LCLs. This finding is consistent with the general observation among all 19 human populations screened in this study of a specific enrichment in very low-levels heteroplasmies and, in a mirrored fashion, quasi-homoplasmies (see Figure 3 and Additional File 10). As indeed demonstrated for both pathogenic predicted and LCL/blood variants analyzed, the majority of low-levels heteroplasmies are unshared between individuals and also associated with very low variability, thus more likely represent a bulk of private variants carried by each individual, as previously suggested [16]. Moreover, in this fashion, our analysis suggests reliability of 1000 Genomes LCL samples and more generally of early passage EBV-transformed lymphocytes for mitochondrial genotyping, although validation with specific molecular methods for the detection of low heteroplasmies [53] and mtDNA targeted resequencing of blood-LCL paired-samples would be recommended.

An interesting finding in our analyses concerned the detection of shared potentially pathogenic variants. These results raise several questions on the definition of pathogenicity for an mtDNA change, a debate that has recently sparked within the mtDNA community and that calls for an in-depth thinking. The pathogenicity parameters assigned to nuclear variants may not apply *sic *to mitochondrial ones, in the same fashion as the concept of polymorphism. Because of the peculiarities of mitochondrial polyplasmic genetics, the assignment of pathogenicity ought to take into account the degree of heteroplasmy, the haplogroup/haplotype context, and even environmental factors. For instance, a report describes a common European variant as predisposing to breast cancer in African women, which puts a red alert sign on the need for clarification in this field [54]. Overall, unless supported by clear-cut functional studies, mtDNA variants ought not to be considered pathogenic independently on the abovementioned factors. These considerations also draw attention on the true existence of a variant. On one side, it appears striking that we detected a large number of previously unreported variants, in a database that contains nearly 10,000 mitochondrial genomes, a large number of which at very low heteroplasmy levels. This may not appear surprising since they may be detected exclusively if highly sensitive methods are applied, such as deep sequencing. Nonetheless, one ought to consider that the increasing sensitivity of sequencing strategies allows the detection of low heteroplasmy degrees, but a limit is reached across which it becomes impossible to distinguish between a true low heteroplasmy and errors, intuitively. From a biological standpoint, extremely low heteroplasmic variants, being these potentially pathogenic or not, may rarely have a physio-pathological meaning (unless they may become amplified during transmission to offspring). We may not assume that all variants here detected in blood-extracted samples were germ-line, as blood is indeed a somatic tissue. With this in mind, one has to ask whether the occurrence of very low variants in a somatic tissue may be more than a completely random transient event, and therefore whether annotation of such variants is a necessary task. Theoretically, every position in the mtDNA may vary in a single copy and be detected before evolution or random drift act to amplify it or to revert it, therefore a consensus must be reached for annotation of very low heteroplasmic data.

Overall, we believe that the accurate annotation of the heteroplasmy degree of mitochondrial variants, which is of paramount importance especially in clinical studies, should be better implemented in up-to-date mitochondrial databases. Indeed, the combined use of several information, such as the health status of individuals, the biological source of DNA, the heteroplasmic status of variants, the prediction of pathogenicity and the haplogroup assignment should be a valuable tool for the definition of general criteria of prioritization of mitochondrial disease-associated variants.

## Conclusions

To the best of our knowledge, this is likely the most extended population-scale mitochondrial genotyping in humans, enriched with the estimation of heteroplasmies. We used a pipeline to extract and characterize mitochondrial genomes from 1000 Genomes Whole Exome Sequencing data, previously disregarded in these individuals. The application of our protocol offers also the relevant opportunity to further use this information, coupled with the nuclear genotype, for nuclear/mitochondrial coevolution studies both in health and disease.

## List of abbreviations

WES: Whole Exome Sequencing; mtDNA: mitochondrial DNA; NumtS: Nuclear Mitochondrial Sequences; indels: insertions and deletions; NGS: Next Generation Sequencing; LCL: lymphoblastoid cell line; GRCh37/hg19: Genome Reference Consortium Human Reference 37/human genome version 19; rCRS: revised Cambridge Reference Sequence; BWA: Burrows-Wheeler Alignment; SAM: Sequence Alignment/Map; QS: quality score; rd: read depth; RSRS: Reconstructed Sapiens Reference Sequence; EBV: Epstein-Barr Virus; BAM: Binary Sequence Alignment/Map; WGS: Whole Genome Sequencing; VCF: Variant Call Format; OriL: origin of light-strand replication; HF: Heteroplasmic Fraction; MELAS: Mitochondrial Encephalomyopathy, Lactic Acidosis and Stroke-like episodes; LHON: Leber's Hereditary Optic Neuropathy.

## Competing interests

The authors declare that they have no competing interests.

## Authors' contributions

Research study was conceived by MAD, CC, GG and MA. Data analyses were carried out by MAD and MS. The bioinformatics pipeline for the extraction of mitochondrial variants, annotation of heteroplasmy and haplogroup assignment was implemented by CC and DS. Additional data analyses were performed by MAD, MS and FMC. Figure and table generation was performed by MAD and MS. MA coordinated and supervised the whole project. MAD, CC, GG and MA drafted the manuscript and all authors read and approved the final manuscript.

## Supplementary Material

Additional File 1 **Samples annotation integrated with haplogroup assignment, mitochondrial coverage and *per *base depth**. Information about ethnicity, gender, mitochondrial genome coverage and the capture technology used for the 1242 samples, is available at the 1000 Genomes web site [26]. Haplogroup for each sample is also reported, predicted through the *mt-classifier *tool in HmtDB [18]. The 1000 Genomes Phase 1 data collection included 827 out of 1242 samples, while the remaining 415 are additional individuals, listed within the 2012-05-22 exome alignment index [27], which comprised all the exome samples aligned by the Consortium. Twenty-eight low coverage samples were used as controls to validate mitochondrial variants detected in exome samples. Click here for file

Additional File 2 **List of mitochondrial variants identified**. All the mitochondrial variant positions and related reference and variant alleles identified within 1242 exome samples are listed in a BED format file, reporting 0-based start and 1-based end coordinates. Click here for file

Additional File 3 **Depth of coverage of 723 Agilent samples and 515 NimbleGen samples**. For each analyzed sample the median depth of coverage was estimated within each mitochondrial locus, starting from *per *base depth values. A better efficiency in mitochondrial reads extraction was obtained with the Agilent (A) kit of enrichment, as suggested by comparing the highest mitochondrial depth values obtained through the Agilent (274.57X) protocol with that reached by the NimbleGen (B, 55.67X) samples within the same locus, *MT-ND6. MT-TS1 *(64.99X) and *MT-TN *(26.44X) show the lowest depth through Agilent and NimbleGen capturing, respectively. Click here for file

Additional File 4 **Quality score of 723 Agilent samples and 515 NimbleGen samples**. Median quality score was estimated for each analyzed sample within each mitochondrial locus starting from quality score of single positions. Agilent (A) mitochondrial reads show slightly higher QS than NimbleGen (B): both capture technologies recorded the maximum score within the same locus, *MT-TS2 *(Agilent 35.82, NimbleGen 33.13), while the lowest value is within *MT-TR *(34.30 with Agilent, 31.47 with NimbleGen). Moreover Agilent QSs seem to have a more uniform trend than Nimblegen ones. Click here for file

Additional File 5 **Variants detected by whole genome low coverage and exome sequencing**. The whole set of variant sites identified within 28 analyzed exome samples is here compared with that detected within the same samples obtained through whole genome low coverage sequencing by the 1000 Genomes Consortium. Each mutation identified by the two methods is reported, integrated with information about homoplasmic/heteroplasmic state within the samples. For 1282/1376 variants, heteroplasmic fractions (HF) estimated by our pipeline are shown, while depth of coverage within exome samples is reported for each position. Click here for file

Additional File 6 **Indels identified in assembled mitochondrial genomes**. The total number of heteroplasmic and homoplasmic indel events (149 deletions and 66 insertions) recorded within the analyzed dataset is here reported. The frequency of each event was estimated considering the dataset size (1242 samples). Fifty-three percent samples display an insertion in the same position, namely the *m.310insC(n)*. Over half of the total events was not carried by more than one sample. The previously reported annotation of these events in healthy and patients was made after consulting HmtDB [18] and Mitomap [17] databases. Click here for file

Additional File 7 **Spectrum of mitochondrial mutations within the 1242 analyzed samples**. The number of mitochondrial variants identified in each sample is here reported. A sorting was made on the heteroplasmic/homoplasmic state and mutation type. Average values and statistical tests results are reported at the end of the table. The mean number of variants was estimated within the whole dataset and also within the two subsets (blood and LCLs). P-value of significance referred to each class of variants (insertions, deletions, mismatches) compared between blood and LCLs. Minimal differences were detected between the two types of samples, especially in relation to indels (p < 0.05). The differences between mean values of heteroplasmic variants per sample in LCLs (7.42) and in blood (7.33) were not statistically significant. Click here for file

Additional File 8 **Indels found within homopolymeric stretches**. Almost all of the identified indels (72.09%) occur within homopolymeric stretches, defined as regions with the same nucleotide in at least two adjacent positions. The figure shows how indels are distributed on the basis of homopolymers length. The shortest homopolymers harbor the highest number of indels, although 5-bases stretches present high levels of both deletions and insertions too. Low frequency of indel events was observed within G-stretches. Click here for file

Additional File 9 **Mismatches identified in assembled mitochondrial genomes**. The total number of heteroplasmic (2330) and homoplasmic (2204) mismatches events recorded within the analyzed dataset is here reported. The number of samples harboring a specific mismatch in a heteroplasmic or homoplasmic state is also shown. About 84% samples display a mismatch in the same position (*m.263A>G*), explained by the fact that this event is defined as one of the seven rare rCRS polymorphisms. Over half of the total events was not harbored by more than 1 sample. The annotation of these events in healthy subjects and patients was made using data annotated in HmtDB [18] and Mitomap [17] databases. Click here for file

Additional File 10 **Hierarchical clustering of heteroplasmic fractions**. A hierarchical clustering based on Euclidean distance was performed to evidence shared classes of heteroplasmy within individuals with the aim to identify a possible clusterization of samples belonging to the same population sharing a similar number of variants referred to the same heteroplasmic range. There is a substantial preponderance of homoplasmic variants (1.00), quasi-homoplasmies (0.90-0.99) and low-level heteroplasmies (0.01-0.10). The white area corresponds to samples carrying at most 1 variant, due to a poor mitochondrial genome coverage. The crowding of "leaves" in the lowest levels of the tree highlights lack of correlation among heteroplasmic clusters and the sample geographical origin. Click here for file

Additional File 11 **Non-synonymous changes within analyzed samples**. After the haplogroup assignment, 543 non-synonymous variant positions were detected, which do not define the individual haplogroup. Among them, 339 were found as homoplasmic and 309 as heteroplasmic. Heteroplasmic fraction (HF) of missense mismatches and samples ethnicity are reported. Click here for file

Additional File 12 **Functional annotation of non-synonymous mismatches in 943 samples**. Predictions of pathogenicity were carried out on 543 non-synonymous mismatches previously filtered, using MutPred [20], Polyphen-2 [21] and SNPs&GO [22] tools. Localization of mismatches, the homoplasmic/heteroplasmic state, the corresponding aminoacid change and nucleotide variability values estimated on nearly 10,000 mitochondrial nucleotide multi-aligned sequences, downloaded from [33], are reported. Above all, *MT-ND5 *appeared to harbor the highest frequency of non-synonymous changes. All the variants predicted as probably pathogenic by at least one of the abovementioned software (148 homoplasmic and 183 heteroplasmic) show very low variability (mean value = 0.02 for homoplasmic mutations, 0.03 for the heteroplasmic ones). Only 14 non-shared mismatches were predicted as damaging mutations by the three software, and among them the *m.3946G>A *was also annotated in Mitomap [17] as associated to MELAS. Click here for file

## Declarations

Publication of this article was funded in part the corresponding author (MA).

This article has been published as part of *BMC Genomics *Volume 15 Supplement 5, 2014: Italian Society of Bioinformatics (BITS): Annual Meeting 2013: Genomics. The full contents of the supplement are available online at http://www.biomedcentral.com/bmcgenomics/supplements/15/S3
