# Supplementary figures and images for: Extraction and annotation of human mitochondrial genomes from 1000 Genomes Whole Exome Sequencing data
Source: BMC Genomics. 2014 May 6;15(Suppl 3):S2. doi: 10.1186/1471-2164-15-S3-S2 (PMC4083402; doi:10.1186/1471-2164-15-S3-S2)

Coverage

A

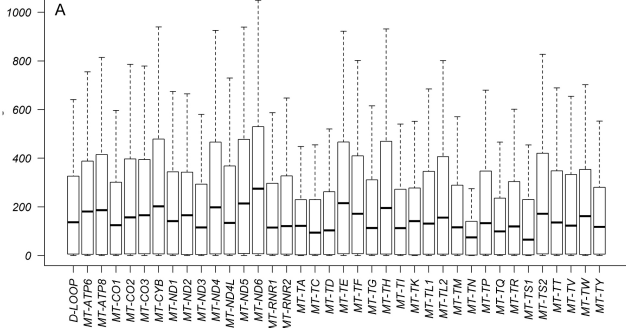

Coverage

B

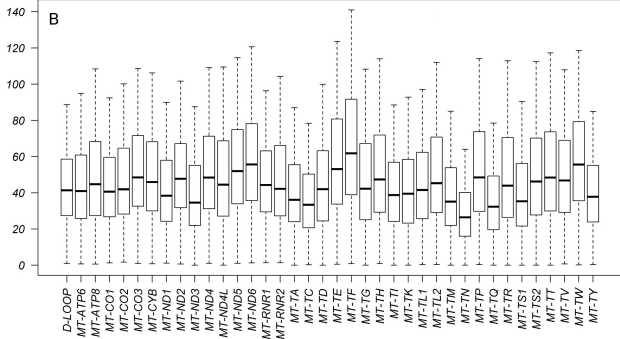

Mitochondrial Loci

Supplement: Additional File 3 — Depth of coverage of 723 Agilent samples and 515 NimbleGen samples. For each analyzed sample the median depth of coverage was estimated within each mitochondrial locus, starting from per base depth values. A better efficiency in mitochondrial reads extraction was obtained with the Agilent (A) kit of enrichment, as suggested by comparing the highest mitochondrial depth values obtained through the Agilent (274.57X) protocol with that reached by the NimbleGen (B, 55.67X) samples within the same locus, MT-ND6. MT-TS1 (64.99X) and MT-TN (26.44X) show the lowest depth through Agilent and NimbleGen capturing, respectively. [file 1471-2164-15-S3-S2-S3.pdf]

Quality Score

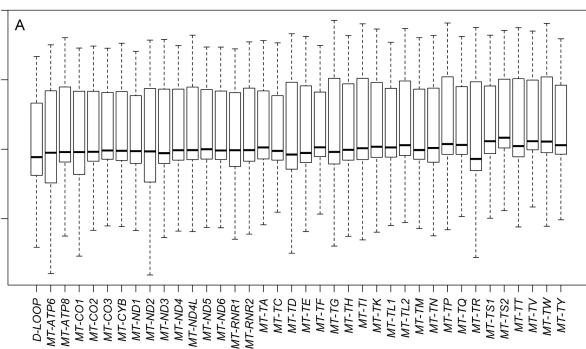

Quality Score

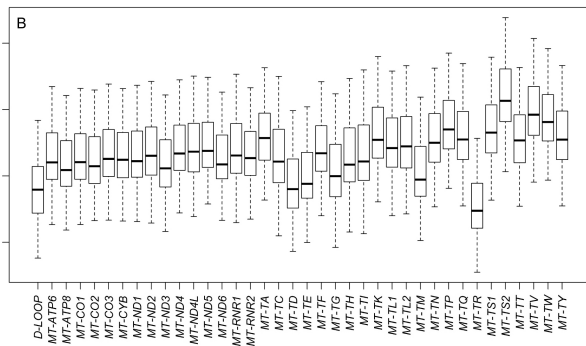

Mitochondrial Loci

Supplement: Additional File 4 — Quality score of 723 Agilent samples and 515 NimbleGen samples. Median quality score was estimated for each analyzed sample within each mitochondrial locus starting from quality score of single positions. Agilent (A) mitochondrial reads show slightly higher QS than NimbleGen (B): both capture technologies recorded the maximum score within the same locus, MT-TS2 (Agilent 35.82, NimbleGen 33.13), while the lowest value is within MT-TR (34.30 with Agilent, 31.47 with NimbleGen). Moreover Agilent QSs seem to have a more uniform trend than Nimblegen ones. [file 1471-2164-15-S3-S2-S4.pdf]

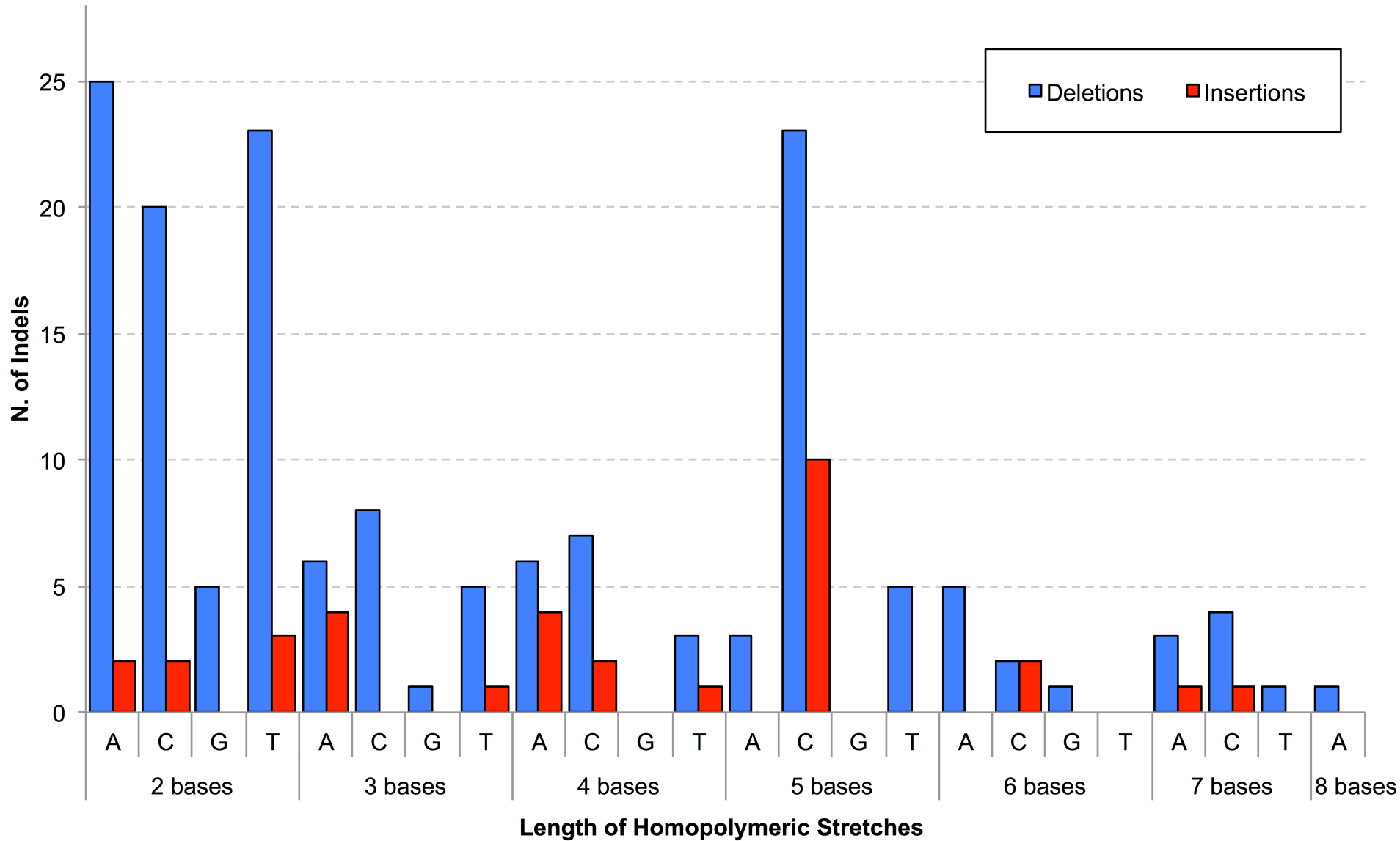

Supplement: Additional File 8 — Indels found within homopolymeric stretches. Almost all of the identified indels (72.09%) occur within homopolymeric stretches, defined as regions with the same nucleotide in at least two adjacent positions. The figure shows how indels are distributed on the basis of homopolymers length. The shortest homopolymers harbor the highest number of indels, although 5-bases stretches present high levels of both deletions and insertions too. Low frequency of indel events was observed within G-stretches. [file 1471-2164-15-S3-S2-S8.pdf]

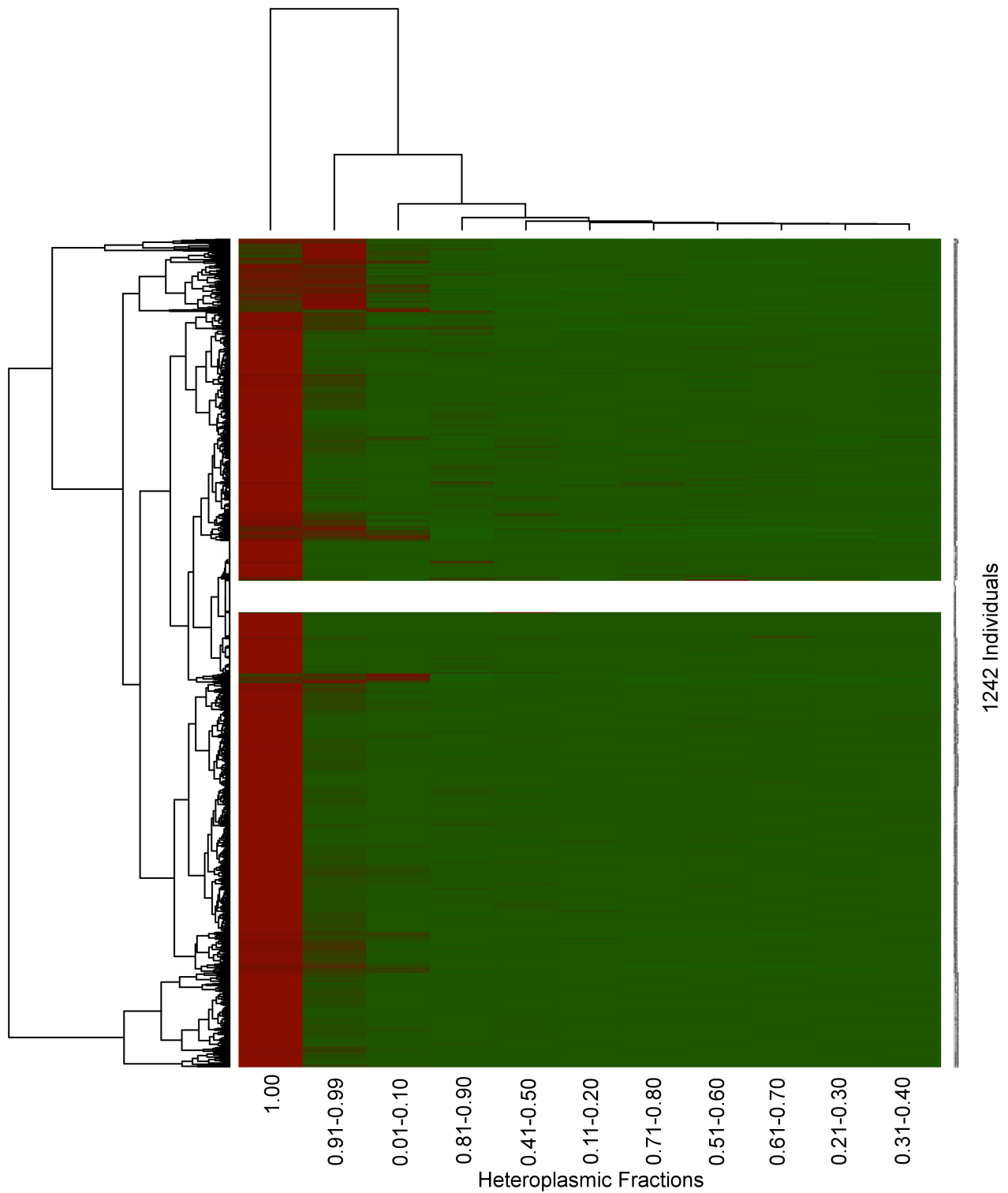

Supplement: Additional File 10 — Hierarchical clustering of heteroplasmic fractions. A hierarchical clustering based on Euclidean distance was performed to evidence shared classes of heteroplasmy within individuals with the aim to identify a possible clusterization of samples belonging to the same population sharing a similar number of variants referred to the same heteroplasmic range. There is a substantial preponderance of homoplasmic variants (1.00), quasi-homoplasmies (0.90-0.99) and low-level heteroplasmies (0.01-0.10). The white area corresponds to samples carrying at most 1 variant, due to a poor mitochondrial genome coverage. The crowding of "leaves" in the lowest levels of the tree highlights lack of correlation among heteroplasmic clusters and the sample geographical origin. [file 1471-2164-15-S3-S2-S10.pdf]
